# Treatment with Idelalisib in Patients with Relapsed or Refractory Follicular Lymphoma: The Observational Italian Multicenter FolIdela Study

**DOI:** 10.3390/cancers14030654

**Published:** 2022-01-27

**Authors:** Beatrice Casadei, Lisa Argnani, Alessandro Broccoli, Caterina Patti, Piero Maria Stefani, Antonio Cuneo, Gloria Margiotta Casaluci, Carlo Visco, Guido Gini, Fabrizio Pane, Francesco D’Alò, Debora Luzi, Maria Cantonetti, Samantha Pozzi, Gerardo Musuraca, Chiara Rosignoli, Annalisa Arcari, Sofya Kovalchuk, Monica Tani, Maria Chiara Tisi, Mario Petrini, Vittorio Stefoni, Pier Luigi Zinzani

**Affiliations:** 1 IRCCS Azienda Ospedaliero, Universitaria di Bologna, Istituto di Ematologia “Seràgnoli”, 40138 Bologna, Italy; bea.casadei@gmail.com (B.C.); alessandro.broccoli@studio.unibo.it (A.B.); vittorio.stefoni2@unibo.it (V.S.); 2 Dipartimento di Medicina Specialistica, Diagnostica e Sperimentale, Università di Bologna, Via Massarenti 9, 40138 Bologna, Italy; lisa.argnani@unibo.it; 3 Division of Onco-Hematology, Azienda Villa Sofia-Cervello, 90146 Palermo, Italy; k.patti@villasofia.it; 4 Hematology Unit, General Hospital Ca’ Foncello, 31100 Treviso, Italy; pieromaria.stefani@aulss2.veneto.it; 5 Hematology Section, St. Anna University Hospital, 44121 Ferrara, Italy; cut@unife.it; 6 Division of Hematology, Department of Translational Medicine, University of Eastern Piedmont and AOU Maggiore della Carità, 28100 Novara, Italy; gloria.margiotta@med.uniupo.it; 7 Department of Medicine, Section of Hematology, University of Verona, 37129 Verona, Italy; carlo.visco@univr.it; 8 Clinic of Hematology, Ospedali Riuniti Ancona, Università Politecnica delle Marche, 60126 Ancona, Italy; guidogini@me.com; 9 UOC di Ematologia e Trapianti di Midollo, Dipartimento di Medicina Clinica e Chirurgia, Azienda Ospedaliera Universitaria Federico II di Napoli, Università di Napoli Federico II, 80131 Napoli, Italy; fabpane@unina.it; 10 Dipartimento di Diagnostica per Immagini, Radioterapia Oncologica ed Ematologia, Fondazione Policlinico Universitario Agostino Gemelli IRCCS, 00168 Roma, Italy; francesco.dalo@policlinicogemelli.it; 11 Dipartimento di Scienze Radiologiche ed Ematologiche, Università Cattolica del Sacro Cuore, 00168 Roma, Italy; 12 S.C. di Oncoematologia, A.O. Terni, Università degli Studi di Perugia, 05100 Perugia, Italy; d.luzi@aospterni.it; 13 Dipartimento di Biomedicina e Prevenzione, Università degli Studi di Roma “Tor Vergata”, 00133 Roma, Italy; cantonet@uniroma2.it; 14 Division of Medical Oncology, Department of Medical, Surgical Science, University of Modena and Reggio Emilia Cancer Center—Policlinico di Modena, 41125 Modena, Italy; samantha.pozzi@unimore.it; 15 IRCCS Istituto Romagnolo per lo Studio dei Tumori (IRST) “Dino Amadori”, 47014 Meldola, Italy; gerardo.musuraca@irst.emr.it; 16 Institute of Hematology, “Santa Maria della Misericordia” Hospital, ASUFC Udine, 33100 Udine, Italy; chiara.rosignoli@edu.unife.it; 17 Unit of Hematology and Bone Marrow Transplantation, Guglielmo da Saliceto Hospital, 29121 Piacenza, Italy; A.Arcari@ausl.pc.it; 18 SOD Ematologia, AOU Careggi, 50134 Firenze, Italy; sofya.kovalchuk@unifi.it; 19 Unit of Hematology, Santa Maria delle Croci Hospital, 48121 Ravenna, Italy; monica.tani@auslromagna.it; 20 Cell Therapy and Hematology, San Bortolo Hospital, 36100 Vicenza, Italy; mariachiara.tisi@aulss8.veneto.it; 21 Department of Clinical and Experimental Medicine, University of Pisa, 56126 Pisa, Italy; mario.petrini@med.unipi.it

**Keywords:** follicular lymphoma, relapsed, refractory, idelalisib, phosphatidylinositol 3-kinase inhibitor

## Abstract

**Simple Summary:**

Idelalisib, the first-in-class phosphatidylinositol 3-kinase inhibitor, approved by the Italian Medicines Agency for the treatment of relapsed/refractory follicular lymphoma patients, showed high antitumor activity with an acceptable safety profile in a phase II registration trial. A 6-year follow-up of the same trial did not reveal any new safety concerns, confirming the role of idelalisib as an effective option for indolent non-Hodgkin lymphoma, refractory to previous therapies. The aim of this multicenter study is to point out the role of idelalisib in a real-life context, since data from everyday clinical practices are scarce. We report the effective and manageable safety profile of idelalisib in the treatment of 72 relapsed/refractory follicular lymphoma patients, bringing further demonstrations of its role in this setting.

**Abstract:**

Follicular lymphoma (FL) is an indolent hematological disease, often responsive to the first line of treatment, but characterized by repeated relapses. The therapeutic algorithm for relapsed/refractory FL patients comprises phosphatidylinositol 3-kinase inhibitors. Idelalisib showed anticancer activity, while inducing a significant rate of toxicities. Since the evidence in the literature on its use in normal clinical practice is scarce, a retrospective multicenter study was conducted to evaluate effectiveness and tolerability in a real-life context. Seventy-two patients with a median age at diagnosis of 57.2 years—mostly with an advanced stage (88.9%) and relapsed to the most recent therapy (79.1%)—were enrolled. The median number of prior therapies was three (20.8% refractory to the last therapy before idelalisib). With a median number of 4 months of treatment, the overall response rate was 41.7% (20.8% complete responses). Median disease-free survival and overall survival were achieved at 8.4 months and at 4 years, respectively. Forty-four percent of patients experienced at least one drug-related toxicity: 6.9% hematological ones and 43% non-hematological. The study confirmed that idelalisib has anticancer effectiveness and an acceptable safety profile in relapsed/refractory FL with unfavorable prognostic characteristics, even in the context of normal clinical practice.

## 1. Introduction

Follicular lymphoma (FL) is the second most frequent lymphoma in Western countries (5–7/100.000 per year), representing about 20% of all non-Hodgkin lymphomas (NHL) and at least 70% of indolent NHL subtypes [1]. Despite its indolent course that leads to an excellent overall survival (OS), FL remains in most cases an incurable disease characterized by multiple relapses that require further treatment. In this setting, much remains to be accomplished in order to identify predictive markers that can guide the choice and sequence of treatments. Chemoimmunotherapy with anti-CD20 antibody (rituximab or obinutuzumab) in combination with anthracyclines or alkylating agents is still the standard first line treatment for patients with advanced-stage and symptomatic FL [2,3,4]. Twenty-four months of maintenance rituximab can prolong progression free survival (PFS), but not OS, in those patients who obtained at least a partial response (PR) with first-line treatment [5].

In relapsed/refractory (R/R) patients, the decision of which therapy to choose is based on: the type of treatments previously received, the response to prior therapies, the age and comorbidities of patients, and the safety and efficacy of the available treatments. In the second-line setting, autologous stem cell transplantation (ASCT) still has a role as a consolidation therapy for young patients who obtained a disease remission with salvage chemo-immunotherapy [6]. For rituximab-refractory patients who are not eligible for ASCT, obinutuzumab plus bendamustine remains a viable option [7]. Recently, lenalidomide in combination with rituximab (R^2^) was approved by the Italian Medicines Agency (AIFA) for the treatment of R/R FL, based on the results of the phase 3 AUGMENT trial, which showed not only a better overall response rate (ORR), but also a better PFS and OS for patients treated with R^2^ in comparison to those treated with rituximab plus placebo [8]. Radioimmunotherapy, such as yttrium-90–labeled ibritumomab, can be a choice in an R/R setting, but its use is granted for patients with an adequate bone marrow function and limited marrow involvement by FL [9].

In recent years, several new therapies such as intracellular pathway inhibitors, CAR T cell therapy, and histone deacetylase inhibitors have received the Food and Drug Administration (FDA) approval for R/R FL; therefore, many more patients can be treated. However, in Italy, only a phosphatidylinositol 3-kinase (PI3K) inhibitor, namely idelalisib, has been approved by AIFA for the treatment of FL patients who have failed at least two prior lines of therapies [10].

PI3K has a catalytic unit with four different isoforms; α and β isoforms are expressed in many tissues, while γ and δ isoforms are predominantly expressed in hematopoietic cells, including B lymphocytes [11]. In particular, a PI3Kδ subunit is essential for B-cell development and function and transducing signals from a B-cell receptor (BCR) [12]. In recent years, it has been shown that the PI3Kδ pathway is also constitutively activated in B-cell malignancies, leading to uncontrolled proliferation and survival of B lymphocytes [13].

Idelalisib is a first-in-class small molecule inhibitor of PI3K that is highly selective for the p110-δ isoform of the catalytic unit. PI3Kδ inhibition blocks downstream cell pathways such as AKT/mTOR, thus promoting apoptosis in B cells [13,14]. In a phase 2 single-arm open-label study (NCT01282424) on 125 patients with refractory indolent NHL, idelalisib showed high antitumor activity with an acceptable safety profile [10,15]. In particular, in the cohort of FL patients (*n* = 72) the ORR was 55.6% (13.9% complete responses (CR) and 41.7 PR) with a median duration of response (mDoR) of 10.8 months. Median PFS was 11 months, meanwhile the median OS was not reached at a median follow-up time of 19.4 months [16]. The most frequent adverse events (AEs) such as colitis, diarrhea, transaminase elevation, and neutropenia were manageable, leading however to the temporary interruption of idelalisib, dose reduction, and discontinuation in 24 (33.3%), 21 (29.2%) and 18 (25.0%) patients, respectively [16]. These results were also confirmed by the data of the UK compassionate use program [17] that pointed out the role of idelalisib in the treatment of high-risk R/R FL patients. However, Bird and colleagues observed higher more serious AEs occurrences, and worse treatment outcomes between Medicare beneficiaries and trial participants aged 65 years or older [18].

Considering the paucity of data regarding the use of idelalisib in a real-life context, we conducted a retrospective Italian multicenter study on the use of idelalisib in everyday clinical practice in order to evaluate its effectiveness and tolerability in patients with R/R FL.

## 2. Materials and Methods

A multicenter observational retrospective study was conducted in 19 Italian hospitals. Consecutive R/R FL patients, who had received at least one dose of idelalisib between December 2014 and January 2018, were enrolled. Eligible patients were older than 17 years, had a confirmed diagnosis of FL grade 1, 2, or 3A, and have failed at least two prior lines of therapy (as per AIFA indication, December 2014). Those patients who already received idelalisib, or any other Pi3K inhibitors as a part of clinical trial, were excluded. A shared database was used after the approval of all the co-investigators, and variables were strictly defined to avoid bias in reporting data.

The local Ethical Committee approved this observational study, as well as our institutional board. All patients signed the informed consent, and we enrolled them consecutively to avoid selection bias. As for the retrospective design of the study, we also received authorization to analyze the data of patients who resulted as deceased or lost to follow-up at the time of data collection. The study was conducted in respect of the Declaration of Helsinki and its later amendments.

The primary endpoint of the study was the effectiveness of idelalisib; defined as the sum of CR and PR rates (ORR). Secondary study endpoints were: PFS, disease free survival (DFS), OS of patients, and the safety profile of idelalisib. OS was calculated from the start of idelalisib to the last follow-up or death by any cause. PFS was calculated from the start of idelalisib to the first disease progression or death. DFS was determined in all CR patients as the time between the first-documented response and the first disease relapse, or death as a result of lymphoma or acute treatment toxicity. Response to the treatment was evaluated by a computed tomography scan (CT scan) and a positron emission tomography scan (PET scan) at baseline, at weeks 12, 24, 36, and 48, and every 24 weeks thereafter during therapy. Following completion of the treatment, PET/CT scans were performed every 6 months for the first 2 years and every 12 months for a further 3 years. In details, PET scans were always combined with CT scans during treatment and during the follow-up in case of relapsed (or suspected relapse); otherwise, during the follow-up only PET scans were performed. Responses were classified according to the International Workshop Criteria for non-Hodgkin lymphomas [19], and we considered 1 cycle equal to 30 days (1 month). Safety and tolerability were assessed by recording type, incidence, and severity of both AEs and treatment-emergent AEs (TEAEs) (assessed with National Cancer Institute Common Terminology Criteria of AEs v4.0). Patients were monitored with blood tests and visits every two weeks for the first 3 months of treatment, and then every month. In the case of TEAE, monitoring was intensified based on severity and clinical condition. Patients’ characteristics and demographics were analyzed with descriptive statistics, and time-to-events functions were estimated with the Kaplan–Meier method. All analyses were conducted with Stata 11 (StataCorp LLC, College Station, TX, USA), and *p* values were set at 0.05.

## 3. Results

### 3.1. Patient’s Characteristics

From December 2014 to January 2018, 72 FL patients received at least one dose of idelalisib (Table 1). At diagnosis, the median age was 57.2 (range 24.5–82.2), 64 patients (88.8%) were in stage III/IV, and 40 had a bone marrow involvement. Forty-seven (65.3%) patients were male, and 94.4% had a good performance status with an Eastern Cooperative Oncology Group (ECOG) performance status score of 0–1. All patients were heavily pre-treated, with a median number of previous lines of therapy of three (range 1–10). Sixty-one patients received rituximab plus an alkylating agent as a first line of therapy. In particular, bendamustine was administered in 35 patients; meanwhile, cyclophosphamide was given to 70.8% patients (35 received cyclophosphamide, doxorubicin, vincristine, and prednisone (CHOP) or a CHOP-like regimen, and 16 received a cyclophosphamide, vincristine, and prednisone (CVP) regimen). Twenty-three patients underwent ASCT. Twenty patients (27.8%) were refractory to first-line therapy, while 15 (20.8%) were refractory to the last therapy received immediately before idelalisib.

### 3.2. Efficacy and Outcomes

**Figure 1:**
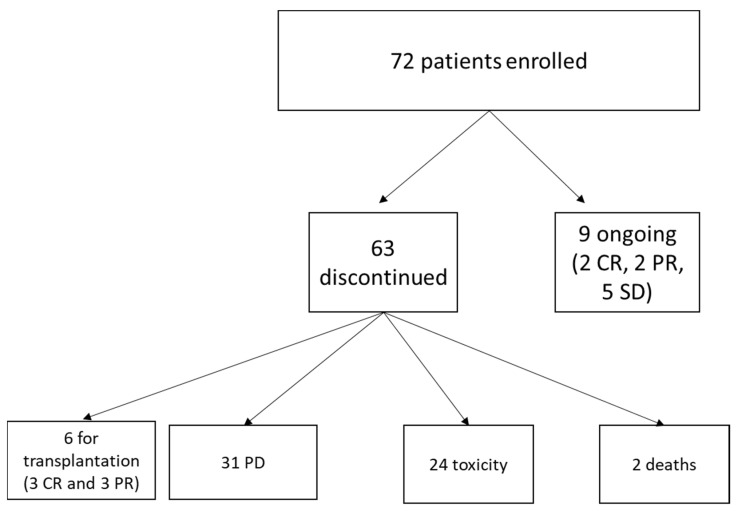
Patient flow. CR—complete response; PD—progressive disease; PR—partial response; SD—stable disease.

**Figure 2:**
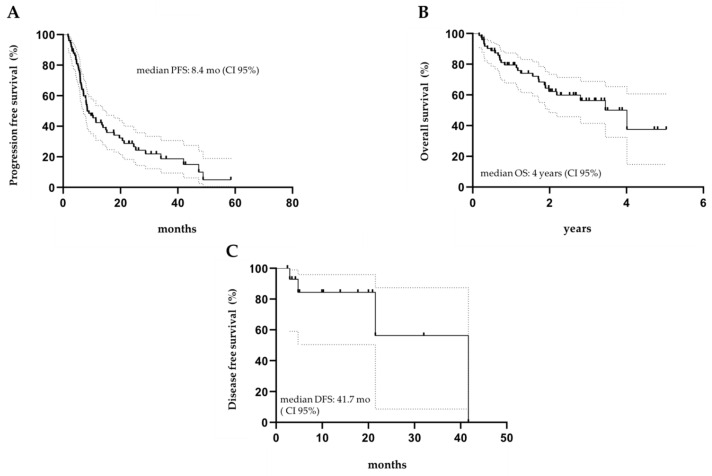
Survival curves of all treated patients: (**A**) median progression-free survival of treated patients; (**B**) median overall-free survival of treated patients; (**C**) median disease-free survival of treated patients.

After a median treatment duration of 4 months (range 1–40), 30 patients obtained at least a PR (15 CR and 15 PR), with an ORR of 41.7%. Responses were achieved quickly, in most cases right after the fourth cycle (21/30, 70%). For those patients in CR, mDoR was 10.2 months (2.5–41.7). There were no differences in terms of responses across different subgroups; in particular, the rate of response was not affected by refractoriness to the previous therapy (*p* = 0.158), age (*p* = 0.722), or sex (*p* = 0.377). Sixty-three (87.5%) patients discontinued the treatment with idelalisib. The main causes of therapy discontinuation were progression of disease (PD) in 31 (43%), and toxicity in 24 (33.3%) patients, respectively. Six patients (three in CR and three in PR), discontinued idelalisib due to transplant consolidation (five had allogeneic-SCT and one received ASCT) (Table 1). At the latest available follow-up, 11 patients (15.3%) were in continuous CR, 8 of whom without further consolidation after a median time of 11 months (at the time of analysis). Twenty-seven patients died: 18 due to PD, the others due to different causes, of whom only one was treatment-related (pneumonia). The flow diagram is reported in Figure 1. The median PFS was 8.4 months (Figure 2A), with a median OS of 4 years, and an estimated OS at 5 years of 37.6% (Figure 2B). Among patients who obtained a CR, four experienced at least a recurrence of the disease, leading to a median DFS of 41.7 months (Figure 2C). The median follow-up time for the whole cohort was 1.7 years (range 3 months–5 years). Thirty-one patients underwent at least one further treatment after idelalisib (mainly transplantation (*n* = 6), bendamustine (*n* = 5), obinutuzumab (*n* = 4), or R^2^ (*n* = 4)), and 11/32 achieved a complete response, which was confirmed at the latest available follow-up at the data cutoff.

### 3.3. Safety

Sixty-one out of 72 patients received idelalisib at the full dose of 150 mg bid; meanwhile, two patients received 100 mg bid, and nine had 150 mg/day (the median dose received was 150 mg bid). The study reflects the real-life context; therefore, the initial dosage was at physicians’ discretion, and depended upon patients’ performance status and comorbidities. In total, 32 (44.4%) patients developed at least one type of toxicity of any grade during the treatment with idelalisib (Table 2). Most of the TEAEs were non-hematological with 43% (*n* = 31) of patients experiencing at least one type of non-hematological toxicity of any grade. The most frequent reported AEs of grade 3 or higher were diarrhea (13.9%), upper respiratory tract infection (13.9%), and pneumonia (6.9%). All patients received antibiotic prophylaxis (trimethoprim–sulfamethoxazole or atovaquone in case of allergy), and no cases of *pneumocystis jirovecii* pneumonia (PJP) were reported (when suspected, a fibro-bronchoscopy with PCR for PJP on the bronchoalveolar lavage was performed to rule out a PJP infection). The most common laboratory abnormalities of grade 3 or higher included elevations in levels of serum alanine or aspartate aminotransferase in three (6.9%) patients. Cytopenias of grade 3 or higher included neutropenia, anemia, or thrombocytopenia in one patient for a total of eight events. One patient developed myelodysplastic syndrome after 4 months of treatment with idelalisib, and died in CR. Due to AEs, 25 of the 61 patients (35.4%), who started at the full dose, had a dose reduction during the treatment. In particular, two reduced due to hematological toxicity, and 23 due to non-hematological toxicity (Table 3). Eighteen out of twenty-five patients, who had dose reduction, permanently discontinued the treatment after a re-challenge due to the recurrence of the AE (Table 3). Patients who were able to continue after dose reduction had toxicities, which were quickly resolved or were easily manageable. AEs led to treatment discontinuation in 24 (33.3%) patients, after a median time of 6.3 months from the start of therapy. Five patients experienced serious AEs (SAE) including: three pneumonia, one diarrhea with dehydration and acute renal failure, and 1 sepsis. Only one patient died due to a SAE (pneumonia). This patient was a primary refractory and had three previous therapies before idelalisib. Infection occurred at the 13th cycle, leading to treatment discontinuation, and the patient died within 1 month during hospitalization.

Due to the retrospective nature of the study, we do not have all of the data regarding the monitoring and management of AEs. Nevertheless, cell counts (and also AST and ALT) were monitored every 2 weeks for the first 3 months of treatment. If a grade 3 or higher neutropenia occurred, a weekly blood cell count was performed. Granulocyte colony-stimulating factor was used for grade 4 neutropenia and for febrile neutropenia of any grade (following AIFA guidelines). The management of a grade 3 diarrhea consisted of: interruption of idelalisib, coprocultures to exclude infectious event, IV hydration, steroid (preferably IV), and possible antibiotic therapy in case of positive coprocultures. For transaminitis the management consisted of: interruption of idelalisib, laboratory workout to exclude hepatotropic virus infection, check after 7 days, and, if grade 3 persisted, treatment with a steroid.

## 4. Discussion

The clinical history of patients with R/R FL is characterized by multiple and increasingly early relapses with the need for different therapeutic strategies, and difficulties in achieving a disease remission; therefore, classifying this setting as an unmet clinical need. In recent years, several new target therapies have been entered in clinical trials, and some of them such as anti-CD19 CAR T-cell therapy, immunomodulatory agents, copanlisib (another PI3K inhibitor), tazemetostat (an EZH2 oral inhibitor), and intracellular pathway inhibitors have been approved by the FDA. In Italy there are few chemo-free regimens approved for R/R patients. In particular, R^2^ for those who have failed at least one previous treatment, and idelalisib for patients refractory to at least two previous lines of therapy.

In our multicenter retrospective study, we analyzed the effectiveness and safety of idelalisib in pre-treated FL, and also confirmed the activity of this treatment in a real-life context.

Our cohort of patients had the same numerosity of the FL cohort treated in the pivotal study [10,16], with 72 patients enrolled; furthermore, the two groups of patients had similar clinical characteristics (Table 1). In particular, they were all heavily pretreated patients (previous median lines: 3 vs. 4 in the pivotal study) with similar frequencies of an ECOG score equal-to or lower-than 1; more than half of them presented with intermediate/high-risk disease (FLIPI ≥ 2), and more than 80% had an advanced stage disease. However, it should be noted that in our cohort only 21% of patients were refractory to the most recent treatment, compared to 86% reported by Salles and colleagues [16]. Thirty out of seventy-two patients obtained at least a PR (ORR 41.7%), with 20.8% (15) achieving a CR. Responses were durable (median 10.2 months) and consistent regardless of patient characteristics, as previously demonstrated [10,15,16,17]. The median PFS was lower than the one reported in the phase 2 trial (8.4 months vs. 11 months, respectively) [10,15,16], but similar to the one reported by Eyre and colleagues in the UK real-life experience (7.1 months) [17]. With a median follow-up time of 1.7 years, four CR patients relapsed, leading to a median DFS of 41.7 months. Although about 50% of patients progressed during therapy with idelalisib, the median OS was reached at 4 years, pointing out how idelalisib may allow for subsequent lines of treatment in our patient cohort. In our study, as well as in the pivotal one, about 87% of patients discontinued the treatment, most of them (43%) due to PD. Six patients in response (three in PR and three in CR), discontinued due to the investigator’s choice to receive transplant consolidation (five had allogeneic SCT and one received ASCT). Three who achieved a CR with consolidation are still in response, underlying the potential role of idelalisib as a bridge to transplantation in patients failing standard chemotherapies [20].

In terms of safety, 44.4% of patients developed at least one type of toxicity of any grade. Even in our experience, the most common TEAEs were non-hematological (43%) with diarrhea; upper respiratory tract infection and pneumonia being the most frequent grade 3 or higher AEs reported. We did not find any cases of PJP, but it must be emphasized that all patients have performed specific antibiotic prophylaxis. Furthermore, all patients were closely monitored for cytomegalovirus (CMV) reactivation, and none required specific antiviral treatment, or had to discontinue due to CMV disease. We thus recommend CMV monitoring in all patients. In particular, for those patients who are CMV-IgG positive before starting idelalisib, CMV viremia should be regularly monitored, at least monthly. Any persistent episode of fever should be monitored, looking for CMV viremia. Compared with what has been reported in the literature, we have had fewer cases of grade ≥ 3 hematological toxicities. This could be explained by close monitoring of blood counts and a greater use of growth factors. AEs led to drug discontinuation in 24 patients, of which 18 had to discontinue for AEs recurrence, despite previous dose reduction. As reported by Wagner and colleagues, patients who underwent idelalisib for one year or more showed longer PFS and OS than those who received therapy for less than one year [15]. Therefore, proper management of toxicities is necessary to increase drug exposure and support favorable outcomes in R/R FL.

Our work outlined the effectiveness of idelalisib in R/R FL patients treated in the context of daily clinical practice. We did not report new safety events, confirming that idelalisib has an acceptable safety profile.

## 5. Conclusions

Idelalisib, the first-in-class oral inhibitor of PI3K δ isoforms, remains the only small and target molecule approved by AIFA for the treatment of refractory FL patients. Since its release, considering the paucity of data coming from everyday clinical practice, some concerns have been raised about its efficacy and safety.

Our Italian multicenter retrospective real-life study confirms that idelalisib represents a valid treatment option for patients with heavily pre-treated FL. To note, a subset of patients can achieve durable responses, although it is not yet possible to identify them in advance. Idelalisib may also have a role as a bridge to transplantation in young and fit patients who have failed standard chemotherapy approaches. In addition, we confirm the toxicity profile of the drug, and stress the need for a careful clinical and laboratory monitoring of patients under treatment.

## Figures and Tables

**Table 1:** Baseline characteristics of enrolled patients.

Characteristics	Present	Salles et al.
	Paper (*n* = 72)	(*n* = 72)
Median age at diagnosis, years (range)	57.2 (24.5–82.2)	NA
Median age, years (range)	63.0 (36.4–84.7)	62 (33.0–84.0)
Sex (male), *n* (%)	47 (65.3)	39 (54.2)
ECOG < 1, *n* (%)	68 (94.4)	66 (91.7)
Stage at diagnosis, *n* (%)		
I/II	8 (11.2)	12 (16.7)
III/IV	64 (88.8)	60 (83.3)
Bone marrow involvement, *n* (%)	40 (55.6)	NA
LDH > UNL, *n* (%)	24 (33.3) *	21 (29.2) °
FLIPI > 2, *n* (%)	41 (56.9)	57 (79.2)
Follicular lymphoma grade 3a, *n* (%)	13 (18.1)	12 (16.7)
Median of previous therapy, *n* (range)	3 (1–10)	4 (2–12)
Previous ASCT, *n* (%)	23 (31.9)	12 (16.7)
Baseline neutropenia	10 (13.9)	8 (11.1)
Baseline anemia	9 (12.5)	8 (11.1)
Baseline thrombocytopenia	7 (9.7)	5 (6.9)
Median time since diagnosis, years (range)	5.8 (0.5–20)	4.7 (0.8–18.4)
Median time since last relapse, months (range)	1.6 (0.2–22.9)	NA
Refractory (≤6 mo) to the first line of therapy, *n* (%)	20 (27.8)	NA
Refractory (≤6 mo) to most recent therapy, *n* (%)	15 (20.8)	62 (86.1)
Relapsed (>6 mo) to most recent therapy, *n* (%)	57 (79.1)	10 (13.8)
Treatment disposition at time of data cutoff, *n* (%)		
Ongoing	3 (4.2)	7 (9.7)
Discontinued:		
PD	31 (43)	38 (52.8)
AE	24 (33.3)	15 (20.8)
Investigator request	6 (8.3) ^§^	4 (5.6) ^£^
Death	2 (2.7)	5 (6.9)
Withdrew consent	0 (0)	3 (4.2)

AE—adverse event; ASCT—autologous stem cell transplantation; FLIPI—follicular lymphoma international prognostic index; ECOG—Eastern Cooperative Oncology Group; LDH—lactate dehydrogenase; mo—months; NA—not available; PD—progression of disease; UNL—upper normal limit; * defined as >248 U/L; ° defined as >234 U/L. ^§^ All six patients in response were referred to stem-cell transplantation, ^£^ including one patient referred to transplant.

**Table 2:** Treatment-emergent adverse events and key laboratory abnormalities.

Event or Abnormality, *n* (%) *	Any	Grade ≥ 3
Diarrhea	10 (23.3)	6 (13.9)
Upper respiratory tract infection	7 (16.3)	6 (13.9)
Increased ALT/AST	6 (13.9)	3 (6.9)
Rash	4 (9.3)	1 (2.3)
Pneumonia	3 (6.9)	3 (6.9)
Neutropenia	3 (6.9)	2 (4.7)
Anemia	3 (6.9)	3 (6.9)
Thrombocytopenia	2 (4.7)	2 (4.7)
Mucositis	2 (4.7)	2 (4.7)
Myelodysplastic syndrome	1 (2.3)	1 (2.3)
Pyrexia	1 (2.3)	-
Vomiting	1 (2.3)	1 (2.3)
Total	43	30

ALT—alanine aminotransferase; AST—aspartate aminotransferase. * % are calculated on the total number of AEs, i.e., 43.

**Table 3:** Treatment-emergent adverse events leading to dose reduction and subsequently to dose discontinuation.

Event or Abnormality	Grade	Onset Time (Cycle)	Permanent Discontinuation	Time of Discontinuation (Cycle)
Vomiting	1	5	No	-
Increased ALT/AST	3	2	Yes	4
Pneumonia	3	2	Yes	9
Diarrhea	3	3	Yes	3
Pneumonia	3	2	Yes	4
Upper respiratory tract infection	3	2	Yes	4
Diarrhea	3	7	Yes	12
Upper respiratory tract infection	2	3	Yes	5
Increased ALT/AST	3	2	No	-
Diarrhea	3	3	Yes	4
Neutropenia	3	2	No	-
Upper respiratory tract infection	3	4	Yes	6
Rash	3	22	Yes	40
Pneumonia	3	2	Yes	3
Upper respiratory tract infection	2	15	Yes	38
Anemia	2	14	No	-
Upper respiratory tract infection	2	3	Yes	33
Increased ALT/AST	2	2	Yes	5
Increased ALT/AST	2	2	Yes	4
Diarrhea	3	4	Yes	7
Rash	2	5	No	-
Increased ALT/AST	2	5	Yes	6
Increased ALT/AST	3	2	Yes	3
Diarrhea	3	3	No	-
Mucositis	3	2	No	-

ALT—alanine aminotransferase; AST—aspartate aminotransferase.

## Data Availability

The data that support the findings of this study are available from the corresponding author upon reasonable request.
